# Antioxidants in Pregnancy: Do We Really Need More Trials?

**DOI:** 10.3390/antiox11050812

**Published:** 2022-04-22

**Authors:** Carolina Di Fabrizio, Veronica Giorgione, Asma Khalil, Colin E. Murdoch

**Affiliations:** 1 Vascular Biology Research Center, Molecular and Clinical Sciences Research Institute, St George’s University of London, London SW17 0QT, UK; carodifabrizio@gmail.com (C.D.F.); giorgione.veronica@gmail.com (V.G.); akhalil@sgul.ac.uk (A.K.); 2 Systems Medicine, School of Medicine, University of Dundee, Dundee DD1 9SY, UK; 3 Fetal Medicine Unit, St George’s University Hospitals NHS Foundation Trust, London SW17 0QT, UK

**Keywords:** oxidative stress, pregnancy, preeclampsia, antioxidants

## Abstract

Human pregnancy can be affected by numerous pathologies, from those which are mild and reversible to others which are life-threatening. Among these, gestational diabetes mellitus and hypertensive disorders of pregnancy with subsequent consequences stand out. Health problems experienced by women during pregnancy and postpartum are associated with significant costs to health systems worldwide and contribute largely to maternal mortality and morbidity. Major risk factors for mothers include obesity, advanced maternal age, cardiovascular dysfunction, and endothelial damage; in these scenarios, oxidative stress plays a major role. Markers of oxidative stress can be measured in patients with preeclampsia, foetal growth restriction, and gestational diabetes mellitus, even before their clinical onset. In consequence, antioxidant supplements have been proposed as a possible therapy; however, results derived from large scale randomised clinical trials have been disappointing as no positive effects were demonstrated. This review focuses on the latest evidence on oxidative stress in pregnancy complications, their early diagnosis, and possible therapies to prevent or treat these pathologies.

## 1. Introduction

During pregnancy, both the mother’s and baby’s health have major implications on a successful pregnancy, and deterioration in either or both can lead to a variety of life-threatening complications. A pre-existing—known or unknown—maternal condition with little impact on maternal health prior to gestation can suddenly induce significant implications during pregnancy (pre-gestational pathology). Alternatively, at the time of conception, the maternal dysfunction may be absent and thus arise and develop during the gestational period (gestational pathology). Regardless of this distinction, more than 500,000 maternal deaths occur worldwide every year due to pregnancy complications and this figure remains persistently high. Furthermore, for every woman who dies from causes related to pregnancy and childbirth, there are another 20 who suffer from pregnancy-related pathologies or who experience other serious consequences (UNICEF, 2009). In 2017, the global incidence of maternal disorders was estimated to be almost 80 million cases [1].

Among maternal diseases, preeclampsia, a pregnancy complication characterized by high blood pressure and signs of damage to other organs or systems after 20 weeks of gestation (Mayo Clinic), is one of the main causes of maternal mortality, resulting in more than 50,000 deaths annually worldwide [2]. In addition, it has been associated with an increased risk for the mother and her child to develop cardiovascular complications and diabetes mellitus later in life [3].

These figures cause a significant increase in the cost in health systems. In 2008, an average complicated pregnancy in the USA was associated with a total maternal cost of USD 27,803, which represents an increase of USD 8000 over an uncomplicated pregnancy [4]. However, these figures do not take into account long-term health implications associated with the disease.

In the United Kingdom (UK), hypertensive disorders of pregnancy (HDP) account for 20% occupancy of antepartum hospital admissions, 25% of intensive care unit admissions, and 20% occupancy of neonatal intensive care units [5].

The placenta represents the pivotal organ for two of the main pregnancy complications: preeclampsia and foetal growth restriction (FGR). Despite being the target of research for the last decades, complete understanding of the placenta remains unclear and pregnancy complications derived from failure of this organ still represent an important cause of maternal and foetal morbidity and mortality worldwide, even in industrialized countries [1]. The human placenta is a transitory organ with a remarkable complexity. Its development begins in the first weeks of pregnancy and does not end until the second trimester, involving two different but related processes: vascularisation to establish a foeto-placental vascular network and a posterior invasion of the maternal spiral arteries, transforming them from a high-resistance low-flow vessel into a low-resistance, high-flow type [6]. During this transition, the placenta moves from a low oxygen/hypoxic environment to an oxygenated one.

Various alterations in the placentation process can have serious consequences during pregnancy and women presenting specific characteristics, including obesity, advanced maternal age, cardiovascular dysfunction, and metabolic syndrome, which are also related to endothelial damage, are at higher risk [7,8]. In this pathological placentation, oxidative stress (OS) plays a major role. Some of the most serious complications associated with pregnancy, such as preeclampsia, FGR, or gestational diabetes mellitus (GDM), show an increase in parameters of inflammation and OS in both the mother and the foetus [9], as well as lower antioxidant status and endogenous antioxidants. These alterations can be observed before the clinical development of such pathologies. When the foeto-placental unit is poorly perfused, free radicals are realised and generate oxidative damage in placental cells; thus, OS can induce DNA damage [10], alter proteins and lipids from the cell membranes [11], and promote peroxidation of low-density lipoproteins. In consequence, antioxidant supplements have been studied as a possible therapy. Unfortunately, results derived from large-scale randomised clinical trials have been rather disappointing. Antioxidants such as vitamins C and E given during pregnancy have not demonstrated any significant positive effects [12,13].

The aim of the present review is to outline the available and newest data on the effects of OS in complicated pregnancies by HDP (including preeclampsia and FGR) or GDM, their early diagnosis, and possible therapies to prevent or treat these pathologies.

## 2. Oxidative Stress in Pregnancy

Oxidative stress is caused by an imbalance between production and accumulation of reactive oxygen species (ROS) in cells and tissues and the ability of a biological system to detoxify these reactive products [14].

ROS can be classified in two groups:

- Free radicals (superoxide, the hydroxyl radicals, lipid peroxy-radicals, and alkoxy radicals);
- Non-radical products (including hydrogen peroxide, peroxynitrite, and hypochlorous acid).

At the cellular level, mitochondria are the major site where the largest amount of ROS is generated through the electron transport chain, formed mainly by four protein complexes. Nicotinamide adenine dinucleotide phosphate hydrogen (NADH) and flavin adenine dinucleotide (FADH2) begin the chain by donating their electrons to complex I and complex II, respectively. These electrons are passed to the following complex in the chain and, by this mechanism, electrons become continuously oxidized and reduced, generating an electron current. 

In pregnancy, ROS are generated mainly in the placenta, but xanthine oxidase (XO) are present in the vascular endothelium, and nitric oxide synthase (NOS) are also sources of ROS [15]. OS mediated by ROS is a common feature of a range of interrelated pathologies and several gestational disorders; excessive ROS in the endothelium can inhibit the expression and function of endothelial nitric oxide synthase (eNOS), thus affecting normal vasodilation and becoming an important factor in the pathogenesis of preeclampsia. They also act as a trigger for atherosclerosis, which plays an important role in cardiovascular diseases. Atheromatous plaque formation results from an early endothelial inflammation, which recruits macrophages and leads to ROS generation. Then, circulating lipoproteins are oxidized by ROS, leading to foam cell formation, lipid accumulation, systemic inflammation, and endothelial dysfunction.

In addition, OS can affect the kidneys with similar mechanisms to atherosclerosis, as evident in the renal damage of preeclamptic patients. ROS production leads to an initial inflammatory stage; however, if the stress continues, the result is the formation of abundant fibrotic tissue that impairs organ function [14].

In diabetic patients, ROS can be induced by hyperglycaemia through mitochondrial respiratory chain enzymes, including XO, NOS, and peroxidases, among others [7]. Furthermore, increased ROS levels can damage macromolecules and lead to cellular senescence and accelerated aging. In a normal environment, telomeres (repetitive sequences of non-coding DNA at the end of a chromosome) protect the chromosome from damage and become shorter each time a cell divides. Eventually, the telomeres become so short that the cell can no longer divide. OS is the most common mechanism underlying accelerated telomere shortening, leading to a number of pathologies including cancer, cardiovascular diseases, diabetes, obesity, and atherosclerosis [16].

In contrast, an antioxidant is “any substance that protects cells from the damage caused by free radicals’’ (National Institute of Health). They are present throughout the organism and can be categorised as follows.

- Enzymatic antioxidants are the first line of defence against ROS. The superoxide dismutase family is composed of manganese superoxide dismutase (MnSOD) located in the mitochondrial matrix, as well as Cu,Zn superoxide dismutase (SOD1) located in the mitochondrial intermembrane space, cytosol, and extracellular space. They catalyse the dismutation of superoxide anion radical to hydrogen peroxide and molecular oxygen, thus metabolizing ROS to innocuous products [17].
- Non-enzymatic or free radical scavengers can reduce and inactivate existing free radicals. They include vitamins C and E, carotenoids, and glutathione, and also represent the most-studied antioxidant supplements in pregnancy.

When there is imbalance between produced and disposed free radicals, or when there is inadequate amount of antioxidants, the organism is exposed to OS. 

### 2.1. Oxidative Stress in Normal Pregnancies

As the placenta develops, it transits from a hypoxic environment to a more oxygenated setting. The period of placental development is characterised by a low grade of OS, increased circulating levels of oxidised low-density lipoproteins (LDL), and a reduction in total antioxidant capacity (TAC). A recent study by Mannaerts et al. [18] has shown that systemic inflammation also increases with the advance of pregnancy, thus activating maternal endothelial cells and increasing OS. 

ROS are necessary for certain cellular functions, such as mitochondrial or endothelial functions, normally in low and stable levels. Redox signalling is pivotal in many physiological processes, whereby oxidative post-translational modifications induce changes in structural and functional characteristics of molecules, thus modifying signalling processes [19]. However, elevated levels of ROS, as observed in pathologic pregnancies, are associated with adverse outcomes, including tissue and mitochondrial damage and accelerated aging.

**First Trimester**

During the first 6–8 weeks of pregnancy, with the placenta not yet completely attached to maternal circulation, the uterine spiral arteries are blocked by intraluminal cytotrophoblast and a physiological and local hypoxia is generated. In this period, since the syncytiotrophoblast cells do not express the antioxidant mitochondrial superoxide dismutase (SOD), ROS levels are high, inducing the production of factors that regulate cell proliferation and angiogenesis, such as vascular endothelial growth factor (VEGF) and placental growth factor (PlGF), which are abundantly expressed in the placenta [20]. 

The angiogenic signalling pathway is tightly regulated at various levels, such as a soluble Fms-like tyrosine kinase-1 (sFlt-1), acting as a decoy receptor to neutralize the pro-angiogenic effects from VEGF and PIGF. Maternal blood levels of sFlt-1 and PlGF have been analysed extensively [21] and are utilised as key biomarkers during pregnancy (see below). 

**Second trimester**

Unblocking of the spiral arteries begins at 8 weeks and continues until 16 weeks of gestation. While spiral arteries lose their muscular layer and transform into a large vessel with low resistance, an increased oxygen tension is quickly imposed, bringing the associated risks of damage from OS. In non-pathological concentrations, OS stimulates cell proliferation. However, with premature opening (unplugging) of the spiral arteries, the placental growth is prejudiced since there is insufficient antioxidant defence by this time. A deficiency in the early development of the placenta restricts its growth, which can never be catch up [22].

From a chemical point of view, these periods of ischemia followed by reperfusion are associated with the conversion of xanthine dehydrogenase into xanthine oxidase, which is a potent source of superoxide free radicals, thus increasing OS. It has been demonstrated that xanthine oxidase activity is increased in the placenta of women affected by HDP. In addition, a lower production of NO is related to the peripheral vasoconstriction observed in preeclampsia [23].

**Third Trimester**

The third trimester is the period of maximum growth of both the placenta and the foetus, corresponding to a great increase in placental vascular and energetic requests. During this stage, the highly metabolic placenta relies on efficient mitochondrial function to produce the necessary energy. In this scenario, a hypovolemic state could progress to placental hypoperfusion and, therefore, to foetal growth restriction.

**Cardiovascular System**

During pregnancy, the maternal cardiovascular system adapts to the increased demand, resulting in an increase in cardiac output, stroke volume, heart rate, and plasma volume. The heart undergoes significant remodelling to keep up with the demand. The right heart volume is significantly higher and left ventricle mass increases by an average of 40% by the end of the pregnancy [24]. Despite all these modifications, in most cases, maternal blood pressure remains stable, compensated by generalized peripheral vasodilation.

**Figure 1:**
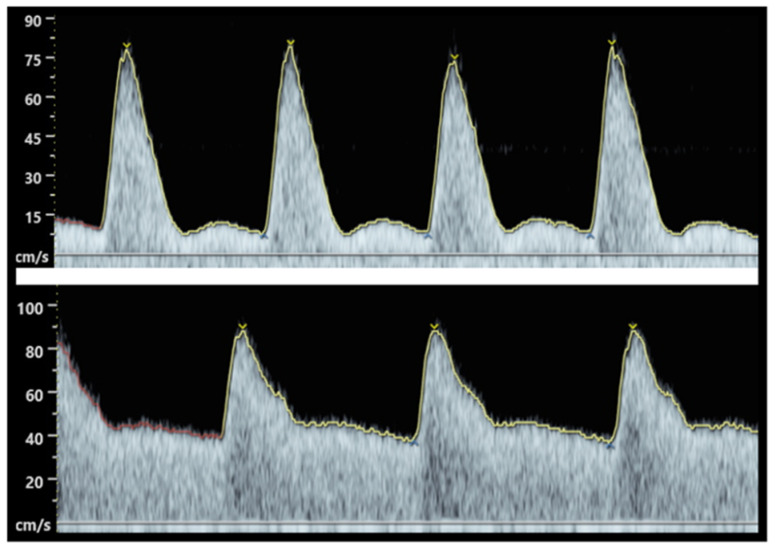
Abnormal (**top**) and normal (**bottom**) uterine artery Doppler waveform in the first trimester. While systolic velocities are similar, the lower waveform show a low resistance vessel with broader systolic peak, as well as a continuous diastolic flow, secondary to a complete remodelling of the spiral arteries. In contrast, the abnormal waveform (**up**) shows an artery with high resistance, as well as a sharp systolic peak, a flow reduction at the start of diastole (“notch”), and low diastolic velocities with poor blood flow.

This vascular change is largely driven by a higher production of NO in endothelial cells [25]. NO is a prime target for inactivation by superoxide and this free radical is increased in preeclamptic pregnancies, thus explaining the generalized peripheral vasoconstriction observed in this pathological state. This systemic vasoconstriction can be clinically observed in preeclampsia and acts as a major indicator of pregnancy complications. In the first trimester, uterine artery Doppler can be performed to assess the risk of preeclampsia. The uterine artery pulsatility index (PI), defined as the difference between peak systolic and end diastolic flow velocity, divided by the time-averaged flow velocity [26], provides a measure of uteroplacental perfusion. A high PI index suggests an increased risk of developing PE, FGR, and stillbirth. Examples of normal and abnormal uterine artery doppler waveform in the first trimester are shown in Figure 1. Moreover, maternal renal vasoconstriction favours hypertension and a modest reduction in glomerular filtration rate with a latter disruption of glomerular fenestrae, causing proteinuria.

### 2.2. Oxidative Stress in Pregnancy Pathologies and Its Biomarkers

Although the main focus of this review is OS damage in pregnancy concerning the foetus and the mother, it is important to note that OS influences the entire reproductive lifespan of women and men. There has been a considerable amount of work regarding oxidative damage in sperm and oocytes. Male gametes are sensitive cells to the accumulation of damaged DNA, which can be induced by a wide variety of factors, such as diet [27], ionization, or even heavy metals [28,29]. Since DNA damage in the gametes could have serious consequences in reproduction [30], the sperm may also play a role in complicated pregnancies.

#### 2.2.1. Oxidative Stress in Preeclampsia

Preeclampsia is a disease in which the mother develops high blood pressure after 20 weeks of pregnancy and presents with proteins in her urine, alterations in blood test, or clinical symptoms (such as severe headache, abdominal pain, or visual alterations). Clinically preeclampsia is defined by American College of Obstetricians and Gynaecologists (ACOG) (2013) as a “syndrome characterized by both new-onset of hypertension plus new-onset proteinuria ≥ 300 mg/24 h after 20 weeks of gestation or, in the absence of proteinuria, hypertension in association with thrombocytopenia, impaired liver function, renal insufficiency, pulmonary edema, or new-onset cerebral or visual disturbances’’ [31]. The only cure available for this clinical entity is delivery, which can represent a major disadvantage for the foetus if the pregnancy is not advanced, adding prematurity to a possible suboptimal development. 

**Figure 2:**
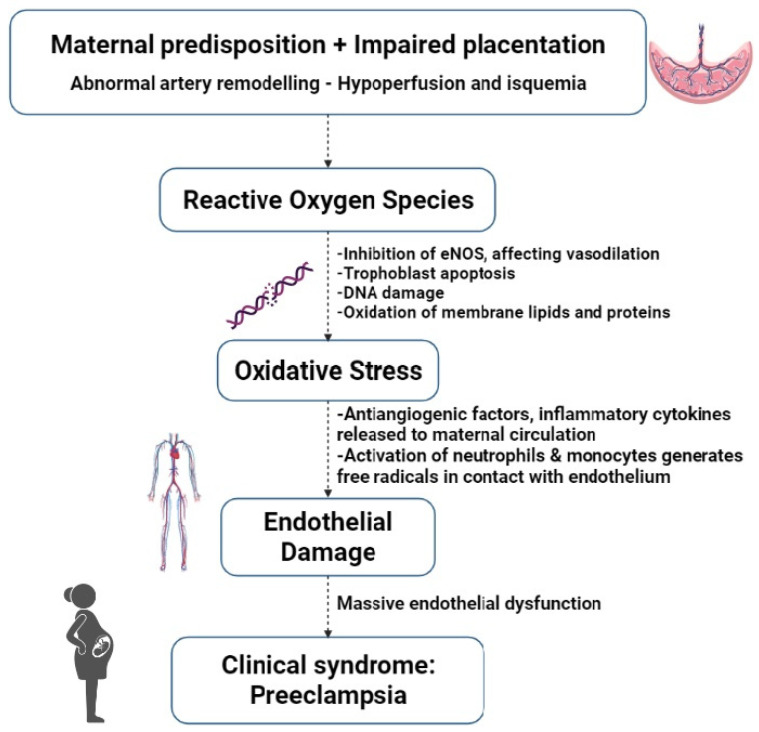
Main events in the development of preeclampsia. eNOS = endothelial nitric oxide synthase.

The aetiology of preeclampsia is not completely understood and has been previously described as a “2 stage-disease” [32]. During the first trimester, abnormal and asymptomatic placentation occurs, followed by a symptomatic maternal syndrome that carries an excess of antiangiogenic factors. The definition was later expanded to a “6-stage” disease [33], incorporating immunological factors (toleration of the mother to the semen of the father), abnormal placentation, and OS in the first 10 weeks, as well as placental damage and atherosis. See Figure 2 for an overview of the main events in the development of preeclampsia. 

According to the timing of clinical onset, before or after 34 weeks of gestation, preeclampsia is classified as early-onset preeclampsia (EoPE) and late-onset preeclampsia (LoPE), respectively. 

EoPE, also referred to as “placental preeclampsia”, is associated with a poor development of the cytotrophoblast early in pregnancy, leading to reduced transformation of spiral arteries. While this subtype is the less frequent (5% to 20% of all cases), its clinical impact is significant, since it can add prematurity to a foetus that has been developing in sub-optimal conditions if delivery becomes necessary. The threshold of foetal viability is around 24 to 26 weeks; however, babies born at this gestational age present high mortality and morbidity, as 50% of neonates that survive will suffer from moderate to severe neurological sequelae [34].

LoPE, also referred to as “maternal preeclampsia”, is associated with a mismatch between the increasing metabolic demands of the placenta–foetal duet and the normal maternal perfusion, contributing to a maternal predisposition to cardiovascular and metabolic pathologies. It represents more than 80% of all cases. Although foetuses at this point are already more developed, they are at risk of being either small or large for gestational age and they tend to have less adaptability and can rapidly deteriorate [35].

Despite the classification of EoPE and LoPE attributing the cause to the placenta and maternal, respectively, it is important to clarify that the combination of both maternal and placental factors can contribute to the development of both types of preeclampsia. 

OS provides one explanation for the pathogenesis of preeclampsia since it leads to lipid peroxidation accompanied by endothelial dysfunction, i.e., a consequence of periods of ischemia–reperfusion generated from failed spiral artery remodelling during placentation. A vicious cycle of enhanced placental OS can allow the release of leukocytes, neutrophils, and cytokines from the placenta, as well as further ROS into the maternal blood circulation, resulting in a massive systemic endothelial dysfunction. In cases where great endothelial damage is observed, arterial compliance is lower and vascular resistance is higher [36]. Furthermore, as widely demonstrated in the Framingham Heart Study [37], arterial stiffness acts as a predictor of cardiovascular disease, cognitive impairment, and dementia.

Elevated ROS levels could also provide the cause for early elevation of antiangiogenic or decrease in angiogenic factors at a time when the placenta needs more vascular development. There is abundant evidence of higher serum and tissue concentrations of biomarkers for OS and systemic inflammation, as well as decreased concentrations of antioxidants, such as vitamin C and vitamin E in women with preeclampsia [38]. 

However, a major challenge in recent decades has been to establish reliable biomarkers for the early detection and prediction of pregnancy complications. Identifying a circulatory biomarker can have major clinical impacts, especially if they can provide early opportunities for clinical intervention. Unfortunately, to date, both predictive and diagnostic biomarkers have rarely been successful. Several molecules related to OS stress have been proposed, since there are direct chemical interactions between ROS and biological components in preeclampsia. Nevertheless, most of these biomarkers are diagnostic, meaning they are elevated by the time the disease is clinically present, thus with scarce predictive capacity. 

Glutathione (GSH), one of the most prevalent antioxidants, can reduce unstable ROS and form oxidized GSH. Reduced GSH represents the most prevalent form of GSH in the organism and the ratio of reduced GSH and oxidized GSH can be used to demonstrate levels of OS. In a study conducted by Kharb et al. [39], women with preeclampsia were found to have significantly lower levels of reduced GSH in blood compared with healthy pregnant controls.

With regards to the lower levels of reduced GSH seen in preeclamptic patients, it is important to note that an association between the use of paracetamol (acetaminophen) and the depletion of GSH has been described [40]. Paracetamol is one of the most consumed drugs during pregnancy, not limited to hypertensive women. In fact, it has been published that more than 40% of pregnant women use paracetamol at least once in pregnancy [41]. Although it is feasible to speculate that paracetamol may be responsible for lower GSH levels, it is doubtful that such a chronic effect would be sustained. Further investigations are needed in order to elucidate the eventual correlation between this worldwide used drug and the antioxidant capacity during pregnancy.

In response to OS, protein carbonyls are generated and researchers have shown elevated levels of these molecules in plasma [42] and placentas [43,44] of preeclamptic women during the clinical stage. However, higher levels of protein carbonyls were also described in pregnancies complicated with GDM [45], making these molecules a poorly specific biomarker. 

#### 2.2.2. Oxidative Stress in Foetal Growth Restriction (FGR)

FGR or intra-uterine growth restriction (IUGR) is a common pregnancy pathology in which the foetus is unable to achieve its genetically determined potential size and weight. Growth restricted foetuses are under the tenth centile of estimated foetal weight compared to standard weights at a given gestational age, affecting 10–15% of pregnancies [46]. They carry a higher risk of long-term complications, such as poor cognitive performance, growth retardation, as well as lifelong risk of cardiovascular disease and metabolic syndrome.

Since the main cause is placental dysfunction, it has been associated with elevated OS. Placentas from pregnancies with FGR have a significantly decreased expression of genes involved in mitochondrial function and oxidative phosphorylation but higher markers of OS [47]. Additionally, elevated levels of oxidized low-density lipoproteins (Ox-LDL) in placental tissue have been shown in patients with preeclampsia and FGR. A major issue with utilising oxidative stress biomarkers for pregnancy complications is that labour activates oxidative stress in the placenta [48], which can lead to wrong conclusions. Care needs to be taken when interpreting results from placenta and blood taken post-delivery. 

Studies that have monitored blood levels of ROS during pregnancies with FGR have shown an increased level of MDA—a breakdown product of lipid peroxidation [49], as well as reduced plasma TAC [50] both in maternal plasma, placental tissue [51], and cord blood in the infants [52]. 

Pregnancy-associated plasma protein-A (PAPP-A) is a protein also related to FGR. It is synthesized by the decidua and measurable in maternal blood in early pregnancy. One of its functions is to cleave IGFBP-4, an insulin growth factor (IGF) inhibitor, thus augmenting the activity of IGFs. Lower levels of PAPP-A are associated with an increased risk for FGR.

#### 2.2.3. Oxidative Stress in Gestational Diabetes Mellitus (GDM)

GDM is usually a transient hyperglycaemic state brought on by pregnancy and linked to insulin dysregulation. Clinically, it is defined as “carbohydrate intolerance of variable severity with onset or first recognition during pregnancy” [53], and it is estimated that, worldwide, one in seven pregnant women may suffer from hyperglycaemia, which in 85% of cases corresponds to GDM (WHO, 2016). However, GDM and type 2 diabetes share a common pathogenesis related to insulin resistance or β-cell dysfunction. It represents an important gestational disorder and a precursor for lifetime disease, since up to half of women with a history of GDM will develop type 2 diabetes five to ten years after delivery [54]. 

Following NICE Guidelines [55], pregnant patients are offered a 75 g 2 h oral glucose tolerance test (OGTT) at 24 to 28 weeks. GDM is diagnosed if the woman has either a fasting plasma glucose level of >5.6 mmol/L or a 2 h plasma glucose level of >7.8 mmol/L. 

Being closely related to obesity, a new term has emerged in recent years: “Diabesity”, considered a modern epidemic, which indicates the coexistence of both diabetes and obesity [23]. In the last three decades, prevalence of GDM has increased ostensibly in all countries despite the income levels (WHO, 2016).

As in preeclampsia, increased ROS and lower plasma antioxidant capacity occur in association with an altered maternal metabolic environment, and GDM is frequently associated with systemic and chronic inflammation. It has been observed that insulin resistance reduces mitochondrial respiration [56] and that human umbilical cord mesenchymal stromal cells from patients with GDM have premature senescence phenotypes and mitochondrial dysfunction. 

The increased levels of ROS are associated with non-enzymatic glycation of macromolecules, producing “advanced glycation end products” (AGEs), which can lead to further OS, inflammatory, and thrombotic reactions, thus playing a role in the development of maternal and neonatal complications [57]. Elevated levels of AGEs are also observed in patients with preeclampsia [58].

Moreover, hyperglycaemia has been found to upregulate NADPH oxidase, whose primary role is to generate ROS [59]. As shown by Leloup et al. [60], more than a decade ago, glucose has the capacity to induce ROS and H_2_O_2_ production in isolated rat islets of Langerhans. Furthermore, insulin secretion can be induced by mitochondrial ROS. It has been demonstrated that incubation of trophoblast from normal placentas, with glucose at a concentration similar to in vivo hyperglycaemic levels, also generates a rise in MDA [47].

Other molecules involved in OS and GDM are F2-isoprostanes, formed mostly by the peroxidation of arachidonic acid. They have an important role in organ vasoconstriction, including in the kidney and the placenta, and can be detected in the plasma and urine of diabetic and preeclamptic pregnant women. Kapustin et al. [61] recently described increased levels of isoprostanes in patients with GDM, thus adding more information to what has already been published by Walsh [62], who had demonstrated higher levels of isoprostanes in preeclamptic placentas. 

### 2.3. Oxidative Stress in Common Risk Factors for Complicated Pregnancies

OS plays an important role, not only in preeclampsia and GDM, but in several disorders which represent risk factors for complicated pregnancy and subsequent (and possible pre-) comorbidities, i.e., type 2 diabetes, renal failure, and cardiovascular disease. 

#### 2.3.1. Endothelial Dysfunction

Vascular endothelium consists of a single but complex layer of epithelial cells covering the interior surface of blood vessels. This organ is responsible for controlling the passage of molecules in and out of the bloodstream; has paracrine and autocrine functions; and plays a role in inflammatory cell adherence, anticoagulation, and angiogenesis. Endothelial dysfunction is a key event in the development of vascular diseases and it is enhanced by OS, leading to cardiac failure, peripheral artery disease, diabetes mellitus, and stroke [47]. It is a state characterized by vasoconstriction, inflammation, and prothrombotic tendency.

On a chemical pathway, excessive production of ROS induces oxidation of tetrahydrobiopterin (BH4), a cofactor of endothelial NOS, which produces NO from l-arginine. In consequence, lower levels of BH4 results in less generation of NO [63]. Moreover, the deficient generation of NO causes systemic vasoconstriction, leading to hypertension, hypoperfusion, and ischemia. In addition, ROS modifies the intracellular influx of calcium, resulting in interstitial oedema, haemoconcentration, and ischemia, as well as further production of ROS, creating a self-perpetuating cycle [47].

Furthermore, IGF-1 is synthesized by several tissues and, after binding with its receptor (IGF-1R), becomes an important mediator of cell growth and differentiation, as well as an antiapoptotic factor in endothelial cells. IGF-1 enhances NO production by eNOS and upregulates antioxidant enzymes, such as glutathione peroxidase (GPX) and superoxide dismutase (SOD). IGF-1-infused animals show lower vascular superoxide levels and higher levels of vascular eNOS and NO [64].

#### 2.3.2. Obesity

The World Health Organization recognizes obesity as a global epidemic. In 2014, approximately 13% of the world’s population was classified as obese, defined as “abnormal or excessive fat accumulation that presents a risk to health. A body mass index (BMI) over 25 kg/m^2^ is considered overweight and over 30 kg/m^2^ is obese” (WHO, 2016). The same organization in 2011 estimated that the female prevalence of overweight and obesity was as high as 77% in the United States, 69% in South Africa, 37% in France, and 32% in China. It is an increasingly common complication in pregnancies, related to the lifestyles of modern society and changes in dietary habits. 

This situation also carries major economic consequences. In 2000, Galtier-Dereure et al. [65] described that the average cost of hospital prenatal care was five times higher in mothers who were overweight before pregnancy than in normal-weight control women.

Obese women are 2–3 times more likely to develop preeclampsia [56] since metabolic factors related to obesity (lipids, insulin, glucose, and leptin) enhance placental and endothelial dysfunction. It has been proposed that obesity induces a state of mitochondrial dysfunction and OS [66]. Furthermore, NO increases blood flow by relaxing the smooth vascular muscle and abdominal, and central obesity leads to an imbalanced production of fat-derived metabolic products, hormones, and adipokines that predispose to a state endothelial dysfunction, activating NADPH oxidase [67]. The FINNPEC cohort [68], a cross-sectional case control study in Finland from 2008 to 2011, confirmed that preeclamptic women had increased pre-pregnancy BMI, demonstrating the close relation between obesity and adverse pregnancy outcomes. 

Obesity also modifies the immune system. The uterine environment of obese women shows natural killer cells overexpressing the Decorin gene, which codifies a protein that promotes the apoptosis of proliferative trophoblasts [69]. 

#### 2.3.3. Advanced Maternal Age (AMA)

Defined as pregnancy among women aged 35 years or older, it has become more prevalent in modern society due to women postponing motherhood, resulting in an advanced maternal age at first pregnancy. In 2013, women aged 35 years or over were responsible for 20% of births in England while women 40 years or over were responsible for 4% of births in England, compared to 6% and 1%, respectively, in 1980 [70]. 

Older women are more likely to suffer from previous pathological conditions, such as obesity, high blood pressure, or insulin resistance, placing them at high risk for pregnancy complications. It has long been suggested that ageing per se is associated with increased inflammation and ROS. As Odame Anto et al. [71] highlighted, women with AMA (35–45 years old) presented higher levels of sFlt-1 at 28–32 weeks and after birth than pregnant women of optimal childbearing age (20–29 years old). Conversely, levels of PIGF, TAC, and PIGF: sFlt-1 ratio were significantly lower in the older group.

## 3. Biomarkers for Prediction of Pregnancy Complications

An important number of clinical trials have been carried out in recent years. Biomarkers for pregnancy complications, possible screening algorithms, and drugs proposed as treatment to these pathologies will be detailed in the subsequent sections. 

### 3.1. Mitochondrial DNA

A molecule recently proposed as a potential biomarker is mitochondrial DNA (MtDNA) which has two main characteristics that make it easy identifiable: it is inherited solely from the mother (hence, results obtained from placenta tissue could be translated for screening using maternal blood) and it is not protected by histones proteins (making it susceptible to OS-induced damage). Studies have shown that there is a significant increase in mtDNA copy number at 15–20 weeks of gestation in preeclampsia cases compared to controls and even higher in EoPE than in LoPE [72,73], possibly as an adaptive response in early gestation to combat the increased OS.

### 3.2. Endothelial Activation

One major advance in biomarkers of endothelial activation in pregnancy has been the use of PlGF and sFlT-1 to predict preeclampsia. As described previously, sFlt-1 is a circulating protein with anti-angiogenic capacity that inhibits PlGF. The latter protein shows lower levels in low oxygen environments and increases along with higher oxygen concentration. In addition, sFlt-1 enhances endothelial dysfunction previously established by OS. What is clinically relevant is that blood levels of PlGF and sFlt-1 are modified in preeclampsia before the clinical manifestation, thus turning these worldwide-used biomarkers into a tool of great clinical utility.

Lower levels of PlGF are seen in preeclamptic patients when compared with normal pregnancies. A recent controlled trial by Duhig et al. [74] detailed that measuring serum PlGF level in women with suspected preeclampsia significantly reduced the time to diagnosis confirmation, thus allowing rapid implementation of treatment and surveillance. The National Institute for Health and Care Excellence (NICE) in the UK recommends use of the sFlt-1/PlGF ratio accompanied by clinical assessment to help predict preeclampsia between 20 and 36.6 weeks. The PROGNOSIS study [25] clearly demonstrated that sFlt-1/PlGF ratio of 38 or lower can be used to rule out the onset of preeclampsia within one week with a negative predictive value higher than 99%. 

Besides the previously detailed biomarkers, endothelial cells synthesize plasminogen-activator inhibitor 1 (PAI-1), a marker of endothelial cell activation. Its concentrations progressively increase during pregnancy and are higher in women with preeclampsia. PAI-2 is synthesised by the placenta and its concentration decreases in preeclampsia due to placental insufficiency. In consequence, PAI-1/PAI-2 ratio is higher in preeclampsia due to endothelial cell activation and placental insufficiency. Although it has been proposed as an effective biomarker, only EoPE (but not LoPE) is associated with increased PAI-1/PAI-2 ratio [75].

### 3.3. First-Trimester Preeclampsia Prediction

The search for robust methods to predict preeclampsia is far from over. However, the use of the Foetal Medicine Foundation (FMF) algorithm can improve first-trimester prediction and diagnosis of preeclampsia. It combines maternal clinical characteristics, angiogenic factors levels (PlGF or PAPP-A), and ultrasound findings (UtA-PI) to estimate risk for preeclampsia. With a false positive rate (FPR) of 5%, this algorithm has a detection rate of 93% for EoPE and 61% for LoPE [76].

A different screening algorithm has been proposed: a combination of placental proteins with MDA. One of those proteins is placental protein 13 (PP-13), expressed in the placenta and released from the syncytiotrophoblast into maternal blood, with lower levels in maternal serum of patients with preeclampsia, but with an increase in the second and third trimesters. Pregnancy-associated plasma protein A (PAPP-A) is another placental protein and its maternal serum concentration is lower in preeclampsia. Beta-HCG is produced by trophoblastic cells of the placenta and promotes angiogenesis, showing higher values in preeclampsia, as well as high levels of MDA. The “Four-parameter combined model”, consisting of MDA+ PP–13 + PAPP-A+ B-HCG, demonstrated a sensitivity and specificity of 97% and 75% in the prediction of preeclampsia in the first trimester [77]. 

In addition, higher levels of oxidative stress and endothelial cell activation in the first trimester could be related to increased arterial stiffness and, therefore, later development of preeclampsia or FGR. Khalil et al. [78] demonstrated that as early as in the first trimester, women who will develop placental insufficiency showed increased arterial stiffness and higher central aortic systolic blood pressure (SBPAo) than women with normotensive pregnancies did. 

After reviewing the theoretical side of oxidative stress in pregnancy, it seems obvious that the answer should be found in antioxidants. For decades, scientists from diverse disciplines have focused their attention on antioxidant drugs or supplements, and numerous trials have been conducted worldwide. Nevertheless, and despite expectations, the results have been disappointing. 

## 4. Antioxidants for Preventing Pregnancy Complications

### 4.1. Vitamins

Vitamins are a group of worldwide used drugs, not only consumed (and commonly recommended) in pregnancy but also in prevention of cardiovascular diseases and cancer, and as a dietary supplement. They have been in the market since the 1940′s and, by 2017, their global market was valued at more than USD 130 billion [79]. These supplements were seen as a promising treatment due to their relative low cost of production and their low incidence of severe adverse effects. In fact, many people think that vitamins may not be efficient, but at least they are safe. Is this true?

Between 2003 and 2005, the multi-centre randomised VIP trial [12], i.e., one of the biggest studies on antioxidants, was performed in the UK. Poston et al. recruited women from 25 hospitals with pregnancies 14 + 0 to 21 + 6 weeks and clinical risk factors for preeclampsia. In total, 2400 participants were given vitamin C (1000 mg) and vitamin E (400 IU) or the placebo from the moment of recruitment until delivery. Blood samples were taken and maternal and foetal outcomes were analysed. Despite showing higher blood concentrations of vitamin C and E, women taking supplements prophylactically did not present a reduction in the rate of preeclampsia and, conversely, the outcomes showed higher risk of gestational hypertension. Moreover, babies born in the interventional group were significantly more likely to have a low birthweight and a lower arterial cord pH at birth. 

The same group published the results of a smaller trial [80] with a similar population, doses, and time of treatment in 1999. PAI-1 and PAI-2 were measured every month until delivery and the PAI-1/PAI-2 ratio was calculated. Vitamin supplementation lowered this biochemical indicator of disease in this population but no clinical differences were observed. Similar results were obtained in 2002 when Chappell et al. [81], using the same drug, dosage, and time of treatment, showed an improvement in biochemical indices of the disease in the control group. However, these trials were not powered enough to assess clinical outcomes. 

In a distinct clinical study conducted in Mexico [82] in 2012, women at high risk of preeclampsia were given energy bars containing l-arginine (a substrate for NO synthesis) and antioxidant vitamins, vitamins only, or the placebo, from 14 to 32 weeks until delivery. The incidence of preeclampsia was significantly lower in the l-arginine + antioxidant vitamins group. However, this study demonstrated that antioxidant vitamins did not show a statistically significant effect by themselves. 

In 2018, Wang et al. [83] published their results of a large case–control study performed in China. Similar to the VIP trial, the study included more than ten thousand women after 20 weeks of gestation. The dietary intake of vitamins C and E, selenium, copper, zinc, and manganese was analysed before or during pregnancy, as well as its relationship with hypertensive disorders. Of concern, a non-statistically significant lower risk of hypertensive disease was found with zinc and selenium taken before pregnancy. No association was demonstrated between vitamin C, vitamin E, copper, or manganese intake and hypertensive disorders. Of note, copper can induce OS also by significantly decreasing glutathione levels [84].

On the contrary, during the same year, Lorzadeh [85] conducted a trial where they studied the effects of vitamins C (1 g/day) and E (400 IU/day) for the prevention of preeclampsia in the second trimester of pregnancy in 160 low-risk nulliparous women. The vitamin group showed a significantly lower incidence of preeclampsia (5% vs. 17.5%), as well as lower mean arterial pressure (MAP). 

One question that might be asked by this point is whether the correct dose was used. The need to adjust the dose of vitamins by maternal weight was recently studied in obese women by Sen et al. [66]. No differences in clinical maternal or neonatal outcomes, nor in any biomarker of inflammation or oxidative stress, could be demonstrated, suggesting that doses may not play a leading role in the effect of antioxidants. 

In fact, Korenc et al. [86] analysed IV vitamin C administration at high doses in postpartum women with severe preeclampsia as a tertiary prevention. They randomized 34 patients to vitamin C (1.5 g within 30 min of delivery and then every 6 h for 72 h) or the placebo, and measured the urinary concentration of biochemical parameters of OS, i.e., N epsilon-(hexanoyl) lysine (HEL), dityrosine, 8-isoprostane, and 8-hydroxy-2-deoxyguanosine (8-OHdg), at first and third day after delivery. The vitamin group showed lower levels of dityrosine and 8-OHdg on day three after delivery. No effect was observed on lipid peroxidation biomarkers.

Several groups have been interested in gathering information and data available in the search of a clearer answer, and systematic reviews and meta-analysis have been published with similar results. As detailed in Table 1, a meta-analysis performed by Conde-Agudelo et al. [87], covering nine studies and more than 19,000 women, analysed the effect of supplementation with vitamin C (1000 mg) and vitamin A (400 UI) to pregnant women at low, moderate, and high risk of preeclampsia. There was no significant difference in the risk of preeclampsia between women receiving supplementation with vitamins C and E vs. those receiving placebo (9.6% vs. 9.6%). Moreover, they found a statistically significant increase in the risk of gestational hypertension, the use of antihypertensive drugs, and the premature rupture of membranes (PROM) among women in the intervention group. No differences were established for foetal or perinatal outcomes.

One year later, a systematic review performed by Salles et al. [88] reached the same conclusion and analysed pregnancy supplementation with antioxidants (selenium, vitamin C, vitamin E, lycopene, and multivitamins). There was no statistically significant difference for preeclampsia, severe preeclampsia, preterm birth, or neonatal death incidence when comparing women receiving antioxidants and the placebo group.

In 2015, two systematic reviews were published, analysing the possible benefits of supplementing with vitamin E or vitamin C in pregnancy to prevent adverse foetal and maternal outcomes. To note, vitamin E interacts synergistically with vitamin C; thus, these supplements are frequently given concurrently. One study included 21 trials with more than 21,000 women [89] and analysed vitamin E supplementation in pregnancy. There was no difference between the groups (supplementation vs. placebo) for the risk of adverse maternal or foetal outcomes, i.e., stillbirth, neonatal death, PE, preterm birth, or FGR. However, vitamin E supplementation was associated with an increased risk of and prelabour rupture of membranes (PROM).

The other systematic review analysed vitamin C supplementation in pregnancy [90]. It included 29 trials (of which 17 are shared with the previous systematic review) and 24,300 women. Similar results were observed, i.e., no clear differences were seen between the intervention group and the placebo group for the risk of stillbirth, neonatal death, perinatal death, birthweight, FGR, preterm birth, preterm or term PROM, and PE. In consequence, the data do not support routine vitamin C or E supplementation in pregnancy.

Finally, in 2018, a meta-analysis conducted by Tenorio et al. [38] investigated the effect of antioxidant supplementation in pregnancy (mostly vitamins C and E). Out of 29 studies analysed, 19 were related to prevention of preeclampsia and 10 studies were related to treatment of the disease, focusing on adverse perinatal outcome. No beneficial effects regarding the prevention of preeclampsia or improvement of perinatal outcomes were observed in this meta-analysis. 

**Table 1:** Nine trials involving a total of 19,810 women were included and randomly assigned to the placebo or vitamin C (1000 mg) + vitamin E (400 IU). Meta-analysis by Conde-Agudelo [87]. PE = preeclampsia.

Author	Year and Country	Participants	GA at Entry (Weeks)	Outcomes
Chappell [80]	1999, UK	283 high-risk pregnant women	16–22	Lower rate of PE in the vitamin group (17% vs. 8%; OR 0·39 [95% CI 0.17–0.90]).
Beazley [13]	2005, USA	100 high-risk pregnant women	14–20	Similar rate of PE in both groups (17.3% vs. 18.8%. RR = 0.92; 95% CI [0.4–2.13]).
Poston [12]	2006, UK	2395 high-risk pregnant women	14–21	Similar incidence of PE in both groups (15% vs. 16%, RR 0.97 [95% CI 0.80–1.17]). In the intervention group, higher incidence of low birthweight babies (28% vs. 24%, 1.15 [1.02–1.30]), but no difference in SGA (21% vs. 19%, 1.12 [0.96–1.31]).
Rumbold [91]	2009, Australia	1877 nulliparous women	14–22	No difference in the risk of PE between groups (6% vs. 5%, RR: 1.20; 95% CI [0.82–1.75]), adverse neonatal outcomes (9.5% vs. 12.1%; RR 0.79; 95% CI [0.61–1.02), or SGA (8.7% vs. 9.9%; RR 0.87, 95% CI [0.66–1.16).
Spinnato [92]	Brazil	707 women with chronic hypertension or history of preeclampsia	12–19	No reduction in the rate of PE (13.8% vs. 15.6%, RR 0.87, 95% CI [0.61–1.25]). No differences in the mean GA at delivery or adverse outcomes. Previous normotensive patients showed a slightly higher rate of severe PE in the study group (6.5% vs. 2.4%, p: 0.11, OR 2.78, 95% CI [0.79–12.62]).
Villar [93] WHO	2009, India, Peru, Vietnam, and South Africa	1355 high-risk pregnant women (previous PE or its complications)	14–22	No association between vitamin supplementation and the rate of PE (RR: 1.0; 95% CI: 0.9–1.3), eclampsia (RR: 1.5; 95% CI: 0.3–8.9), GH (RR: 1.2; 95% CI: 0.9–1.7), low birthweight (RR: 0.9; 95% CI: 0.8–1.1), SGA (RR: 0.9; 95% CI: 0.8–1.1), or perinatal death (RR: 0.8; 95% CI: 0.6–1.2).
Xu [94]	2010, Canada and Mexico	2363 unselected women (later stratified by risk factors for PE)	12–18	No difference in the risk of GH and its adverse outcomes between the groups (RR: 0.99; 95% CI [0.78–1.26]). Vitamin supplements increased the risk of foetal loss, perinatal death, and PPROM.
Roberts [95]	2010, USA	9969 low-risk nulliparous women	9–16	No significant difference between the groups in the rates of GH (6.1% vs. 5.7%; RR 1.07; 95% CI [0.91–1.25]), preeclampsia (7.2% vs. 6.7%, RR 1.07; 95% CI, [0.93–1.24]), or adverse perinatal outcomes.
McCance [96]	2010, UK	761 women with type 1 diabetes	8–22	Similar rates of PE between the groups (15% vs. 19%; RR 0.81, 95% CI [0.59–1.12]).

### 4.2. Melatonin

In addition to being essential in the circadian rhythm, melatonin is a safe and potent antioxidant acting as a direct scavenger of free radicals and indirectly upregulating antioxidant enzymes. It crosses the placental barrier and may have an effect on FGR and brain development. In preeclamptic women, circulating melatonin levels are significantly lower than in healthy pregnancies.

A recent randomized controlled trial [97] on melatonin supplementation with 30 mg/daily versus the placebo was studied, showing a significantly longer interval from preeclampsia diagnosis to delivery in the intervention group. Although no significant change in maternal blood pressure or uterine artery PI was observed, women receiving melatonin required less antihypertensive drugs compared to controls. Moreover, babies in the treated group had a higher rate of being SGA. 

### 4.3. Lycopene

Lycopene is a carotenoid found in several foods worldwide, it does not have pro Vitamin A activity and it has been widely studied for its antioxidant properties. Two trials conducted in India studied the effect of 4 mg lycopene supplementation in preeclampsia and FGR. Treatment was initiated in the second trimester until delivery in women without any medical complication. Sharma et al. [98] recruited 250 primigravida and analysed the effect of lycopene in preeclampsia and FGR, while Antartani and Ashok [99] randomized 54 pregnant women with a high risk for preeclampsia and showed that lycopene could not decrease the incidence of preeclampsia in this population. Both studies showed a lower incidence of growth-restricted babies in the lycopene group; however, only Sharma et al. demonstrated a significantly lower incidence of preeclampsia and FGR in the intervention group. 

Lycopene has not only been studied in HDP. In a large study, Gao et al. [100] demonstrated that lycopene intake during pregnancy is inversely associated with GDM risk, even after adjusting for confounding factors, probably due to its antioxidant characteristics. 

### 4.4. Selenium (Se)

Selenium is a cofactor of the glutathione peroxidase and thioredoxin reductase; thus, its intake might benefit women at risk for IUGR through its capacity as an antioxidant and anti-inflammatory. In a study with 60 high-risk primigravida (due to abnormal uterine artery Doppler waveform), randomly divided (1:1) into two groups to take either 100 μg selenium supplements or the placebo from 17 to 27 weeks of gestation, a higher proportion of women with UtA PI < 1.45 was found in the selenium group when compared with the placebo [101]. This and other main trials performed in the last 5 years are described in Table 2. 

Similarly, in 2014, a UK trial was performed with 230 nulliparous women [102] who were randomized to selenium (60 μg/d) or the placebo for earlier intervention (12 to 14 weeks until delivery). The intervention group showed a higher Se concentration in plasma and a decreased sFlt-1 concentration at 35 weeks as well. 

However, very recently, a large population-based study from Norway demonstrated that there was no association between selenium intake, selenium blood status, and hypertensive disorders of pregnancy in more than 2500 women [103]. 

**Table 2:** Main trials relating antioxidants with gestational hypertensive disorders performed in 2017–2020. PE = preeclampsia. MAP = mean arterial pressure. UtA PI = uterine artery pulsatility index. Mn = manganese. Cu = copper. Zn = zinc.

Author	Country/Year	Participants	Intervention Drug and Daily Doses	Outcome
Sheikhi [104]	Iran, 2017	148 pregnant women	Antioxidants in daily nutrition	No association between intake of antioxidant and risk of PE.
Mesdaghinia [101]	Iran, 2017	60 primigravida women at risk for IUGR	Selenium: 100 μg	Beneficial effects on UtA PI, markers of insulin metabolism, and HDL-C levels. No effect on MDA, NO, and lipid profiles.
Wang [83]	China, 2018	10,228 pregnant women	Vitamin C, vitamin E, Cu, Zn, Se, Mn	Vit C, Vit E, Cu, Mn: No association with hypertensive disorders. Zn and Se: Lower risk of HDP.
Hobson [97]	Australia and Canada, 2018	68 women with PE between 24 and 35.6 weeks	Melatonin: 30 mg	Melatonin group: Higher risk of SGA and proteinuria. Higher interval (6 days) from diagnosis to delivery. Lower need of antihypertensive drugs. No difference on MAP; maternal serum levels of 8-isoprostane, sFlt1, activin A, PlGF, or TAC. No changes in UtA IP.
N. Lorzadeh [85]	Iran, 2020	160 nulliparous women	Vitamin C: 1000 mg + vitamin E: 400 μg.	Intervention group: Lowe incidence of PE. Lower MAP before and after intervention.

### 4.5. Aspirin

Only one drug has been successful in achieving a significant reduction in the incidence of preeclampsia and that is aspirin™ (acetylsalicylic acid). It has been an important drug for more than 120 years due to its pain relief capacity and cardiovascular prevention properties, and it is included in the WHO’s list of essential medications [105]. The aspirin for Evidence-based Preeclampsia Prevention (ASPRE) trial is a world-renowned study published in 2017 [6]. It was the biggest multicentre, prospective, double-blind placebo-controlled randomised trial performed on the prophylactic use of aspirin in pregnancy. By using the FMF combined screening at 11–14 weeks, women at high risk of preeclampsia (>1:100) were identified and randomized to aspirin vs. the placebo. In the aspirin group, the patient received 150 mg per day at bedtime from 11 to 14 until 36 weeks of gestation. The trial showed that in women with singleton pregnancies at high risk of PE, the administration of aspirin resulted in a significantly reduction (62%) of the incidence of EoPE. 

Acetylsalicylic acid is a non-steroidal anti-inflammatory drug that irreversibly inhibits cyclooxygenase 1 and 2 (COX 1 and COX2, respectively), i.e., enzymes that produce prostaglandins and thromboxanes from arachidonic acid. Prostaglandins are synthetized in the endothelium and show vasodilator and anti-inflammatory effects. Oppositely, thromboxanes promote platelet aggregation and vasoconstriction. Nonetheless, given at low doses (150 mg), aspirin selectively inhibits production of thromboxanes, but not prostaglandins, thus decreasing the vasoconstrictive action of thromboxanes.

In addition, it has been shown by in vitro studies that acetylsalicylic acid can increase PlGF production and have an influence in cytokine imbalance in pregnancy [106]. By this mechanism, aspirin can have an anti-inflammatory effect, contributing to endothelium stabilisation.

Finally, research in mice has shown animals receiving low-dose aspirin, as well as higher serum concentrations of antioxidative enzymes (such as SOD) [107].

Despite these explanations, the exact mechanism by which aspirin reduces the incidence of preeclampsia remains unclear.

## 5. Future Directions from Experimental Evidence

Even though antioxidant trials largely turned out to be negative, research is still ongoing to find an optimal antioxidant drug to prevent or treat pregnancy related complications. Focus has been on more selective antioxidants and has investigated intracellular sources of OS during pregnancy. 

**l-Ergothioneine (ERG):** ERG serves as an antioxidant and cellular protectant against various kinds of ROS. Experimental ERG administration in reduced uterine perfusion pressure (RUPP) rat model of preeclampsia [108] showed beneficial effects in RUPP rats, but not in the control group; therefore, it may be useful in patients with preeclampsia in response to placental ischemia, but not in normal pregnancies. No differences in micro-albumin:creatinine ratio, protein:creatinine ratio, or placental weight were observed.

**Mitochondria specific drugs:** Mitochondria and the oxidative stress generated by them are new potential targets that address the vascular dysfunction of preeclampsia. Mitochondria-targeted antioxidant therapies could be an important treatment for adverse pregnancy outcomes, including preeclampsia and FGR. Two main drugs have been studied.

- MITO TEMPO: Mitochondria-targeted superoxide dismutase antioxidant mimetic. It improved endothelial function and reduced mROS production in an in vivo model of hypertension [109], and modified the inflammatory profile of endothelial cells treated with preeclampsia plasma.
- MITO Q: This substance is reduced in the mitochondria to active ubiquinol, which prevents lipid peroxidation and mitochondrial damage. MitoQ has been successfully used in humans, showing a reduced placental MDA and lower levels of placental lipid peroxidation. Interestingly, two main responses have been observed with MitoQ supplementation in mice, depending on the pregnancy period of administration. Although it shows a protective effect against hypertension and kidney damage induced by RUPP rats when administered in late gestation, it exacerbates the preeclampsia-like phenotype when given in early gestation, predisposing to a smaller placenta and increasing proteinuria. As mild OS is required to normal trophoblast proliferation, which occurs due to Mito-Q interference with placenta formation in early pregnancies [110,111]. These findings may help explain the negative results from clinical trials and suggest that more research should be conducted.

Table 3 describes some of the planned trials for the years to come. Despite analysing the possible effect of drugs or biomarkers that have been already studied, they differ by evaluating the effect adjusted by dose or in combination with other substances. As an example, one particular ongoing research is focused on the effect of pravastatin, a statin, in pregnancies with placental insufficiency. Statins are prescribed worldwide due to their capacity of lowering the cholesterol levels; however, additionally, they contribute to endothelial protection through their pro-angiogenic, anti-inflammatory, and antioxidant effects. With preeclampsia being a disease with widespread endothelial damage, we may be facing a novel intervention.

## 6. Conclusions

Designing effective drug treatments for complicated pregnancies, such as preeclampsia and FGR, has been a major challenge in maternal and foetal health for some time. It is essential to ensure an early diagnosis and an efficient treatment since the impact on maternal and foetal health can have long-term consequences. Although there is evidence of elevated OS levels in women with preeclampsia, the role of this mechanism is less clear, raising questions around whether it is a causative or an associated factor in the pathophysiology of preeclampsia. Furthermore, redox homeostasis appears to be crucial for a healthy pregnancy; hence, alternating the balance at an early stage may explain why antioxidants have failed in large-scale clinical trials and, in some cases, had negative effects.

As demonstrated by the aspirin therapy, early therapeutic intervention can help with pregnancy-related complications. In addition, the timing of antioxidant administration, rather than the doses or the type of drug, might be the essential component to assess the management of preeclampsia. Possibly, focusing on antioxidants therapy during the second trimester, as several studies did, may be too late since the critical vascular dysfunction has already occurred.

Despite small randomized trials suggesting that patients with a high supplementation risk of preeclampsia with antioxidants may be beneficial, the overwhelming evidence from large, randomized, and placebo-controlled trials clearly found that vitamin supplementation in pregnancy does not prevent the development of preeclampsia. Moreover, the largest clinical trials included in the meta-analysis—and, in consequence, those with more power in the analysis—used vitamins E and C, i.e., supplements without proven beneficial effects. This detailed feature may explain why the systematic reviews have shown disappointing results.

Millions of US dollars and research resources have passed since the Cochrane review [112] detailed that there was no significant difference between antioxidant and control groups for the relative risk of preeclampsia or any other analysed outcome thirteen years ago. Thus, Talaulikar and Manyonda [113] were arguably right when they suggested that it was time to “give up the ghost” on antioxidants for preeclampsia treatment/prevention twelve years ago. What is more surprising is that even though antioxidant treatment for preeclampsia has clearly failed, future trials involving antioxidant treatment (among other drugs) in complicated pregnancies are planned and several are expected to be completed in subsequent years (see Table 3).

Considering all the high-quality studies and information available, do we really need more trials on antioxidants?

## Figures and Tables

**Table 3:** Ongoing and planned trials involving antioxidant treatment. FGR = foetal growth restriction. SGA = small for gestational age. PE = preeclampsia. OS = oxidative stress.

Study Name	Hypothesis/Objective	Status	Recruitment	Institution	Intention to Publish
Treatment of FGR with l-arginine.	To study if l-arginine in pregnant women with FGR decreases the risk of SGA babies and evaluate perinatal outcomes	Ongoing	No longer recruiting	Hospital Clínico Universitario Virgen de la Arrixaca (Spain)	December 2021
Pravastatin for Pregnancies complicated by Ischemical Placental Disease	To assess the effect of pravastatin in placental insufficiency, the latency period of pregnancy, levels of endothelial factors in the blood, and maternal and neonatal outcomes	Ongoing	No longer recruiting	Aristotle University of Thessaloniki, Greece	December 2021
The Correlation Between Vitamin A/E Levels and PE	To study the correlation between intake of vitamin A, vitamin E, and both together in PE occurrence	Ongoing	Recruiting	School of Medicine, Zhejiang University (China)	January 2022
PLANES-placental growth factor led management of the SGA foetus: a feasibility study	To assess the feasibility of delivering sFlt-1/PIGF ratio led management of women with an SGA foetus, as well as its acceptability to women and clinicians	Ongoing	Recruiting	Dr Jane Harrold. Liverpool Women’s Hospital (UK)	April 2022
Role of Aspirin in Placental and Maternal Endothelial Cell Regulation In PE (ASPERIN)	To determine if aspirin has a dose-dependent response for modifying biomarkers of endothelial dysfunction in women at risk of PE	Ongoing	Recruiting	John O’Brien, MD. University of Kentucky (USA)	June 2022
Antioxidant Use in Diabetes to Reduce OS	To see whether potent and better-targeted antioxidants are successful in preventing birth defects in the offspring of women with diabetes	Ongoing	Active, not recruiting	University of Maryland, Baltimore (USA)	June 2024
Endothelium-dependent Vasodilatation and Other Biomarkers: Predictive Indicators of the Progression From Gestational Hypertension to PE?	To study the correlation between the alteration of endothelium-dependent vasodilatation in pregnant women with stable hypertension with the occurrence of PE	Planned	Not yet recruiting	Avicenne Hospital and Jean Verdier Hospital, Seine Saint Denis, (France)	September 2024

## References

[1] Moran P.S., Wuytack F., Turner M., Normand C., Brown S. (2020). Economic burden of maternal morbidity—A systematic review of cost-of-illness studies. PLoS ONE.

[2] Duley L. (2009). The Global Impact of Pre-eclampsia and Eclampsia. Semin. Perinatol..

[3] Roberts J.M., August P.A., Bakris G., Barton J.R., Bernstein I.M., Druzin M., Gaiser R.R., Granger J.R., Jeyabalan A., Johnson D.D. (2013). Hypertension in Pregnancy. Obstet. Gynecol..

[4] Reuters T. The Cost of Prematurity and Complicated Deliveries to U.S. Employers. Report Prepared for the March of Dimes Foundation. 29 October 2008. https://www.marchofdimes.org/peristats/pdfdocs/cts/ThomsonAnalysis2008_SummaryDocument_final121208.pdf.

[5] Rosenberg K., Twaddle S. (1990). 6 Screening and surveillance of pregnancy hypertension—An economic approach to the use of daycare. Baillière’s Clin. Obstet. Gynaecol..

[6] Rolnik D.L., Wright D., Poon L.C., O’Gorman N., Syngelaki A., Matallana C.P., Akolekar R., Cicero S., Janga D., Singh M. (2017). Aspirin versus Placebo in Pregnancies at High Risk for Preterm Preeclampsia. N. Engl. J. Med..

[7] Fernández-Sánchez A., Madrigal-Santillán E., Bautista M., Esquivel-Soto J., Morales-González A., Esquivel-Chirino C., Durante-Montiel I., Sánchez-Rivera G., Valadez-Vega C., Morales-González J. (2011). Inflammation, Oxidative Stress, and Obesity. Int. J. Mol. Sci..

[8] Nieto M.C., Barrabes E.M., Martínez S.G., Prat M.G., Zantop B.S. (2019). Impact of aging on obstetric outcomes: Defining advanced maternal age in Barcelona. BMC Pregnancy Childbirth.

[9] Hansson S.R., Nääv Å., Erlandsson L. (2015). Oxidative stress in preeclampsia and the role of free fetal hemoglobin. Front. Physiol..

[10] Phaniendra A., Jestadi D.B., Periyasamy L. (2015). Free Radicals: Properties, Sources, Targets, and Their Implication in Various Diseases. Indian J. Clin. Biochem..

[11] Jauniaux E., Poston L., Burton G.J. (2006). Placental-related diseases of pregnancy: Involvement of oxidative stress and implications in human evolution. Hum. Reprod. Update.

[12] Poston L., Briley A., Seed P., Kelly F., Shennan A. (2006). Vitamin C and vitamin E in pregnant women at risk for pre-eclampsia (VIP trial): Randomised placebo-controlled trial. Lancet.

[13] Beazley D., Ahokas R., Livingston J., Griggs M., Sibai B.M. (2005). Vitamin C and E supplementation in women at high risk for preeclampsia: A double-blind, placebo-controlled trial. Am. J. Obstet. Gynecol..

[14] Pizzino G., Irrera N., Cucinotta M., Pallio G., Mannino F., Arcoraci V., Squadrito F., Altavilla D., Bitto A. (2017). Oxidative Stress: Harms and Benefits for Human Health. Oxidative Med. Cell. Longev..

[15] Volpe C.M.O., Villar-Delfino P.H., Anjos P.M.F.d., Nogueira-Machado J.A. (2018). Cellular death, reactive oxygen species (ROS) and diabetic complications. Cell Death Dis..

[16] Barnes R.P., Fouquerel E., Opresko P.L. (2019). The impact of oxidative DNA damage and stress on telomere homeostasis. Mech. Ageing Dev..

[17] Miriyala S., Spasojevic I., Tovmasyan A., Salvemini D., Vujaskovic Z., Clair D.S., Batinic-Haberle I. (2012). Manganese superoxide dismutase, MnSOD and its mimics. Biochim. Biophys. Acta (BBA)—Mol. Basis Dis..

[18] Mannaerts D., Faes E., Cos P., Briede J.J., Gyselaers W., Cornette J., Gorbanev Y., Bogaerts A., Spaanderman M., Van Craenenbroeck E. (2018). Oxidative stress in healthy pregnancy and preeclampsia is linked to chronic inflammation, iron status and vascular function. PLoS ONE.

[19] Lee M.Y., Griendling K.K. (2008). Redox Signaling, Vascular Function, and Hypertension. Antioxid. Redox Signal..

[20] Huang Q.T., Zhang M., Zhong M., Yu Y.H., Liang W.Z., Hang L.L., Gao Y.F., Huang L.P., Wang Z.J. (2013). Advanced glycation end products as an upstream molecule triggers ROS-induced sFlt-1 production in extravillous trophoblasts: A novel bridge between oxidative stress and preeclampsia. Placenta.

[21] Schoots M.H., Gordijn S.J., Scherjon S.A., van Goor H., Hillebrands J.-L. (2018). Oxidative stress in placental pathology. Placenta.

[22] Agarwal A., Aponte-Mellado A., Premkumar B.J., Shaman A., Gupta S. (2012). The effects of oxidative stress on female reproduction: A review. Reprod. Biol. Endocrinol..

[23] Maia L.B., Moura J.J.G. (2018). Putting xanthine oxidoreductase and aldehyde oxidase on the NO metabolism map: Nitrite reduction by molybdoenzymes. Redox Biol..

[24] Melchiorre K., Sharma R., Khalil A., Thilaganathan B. (2016). Maternal Cardiovascular Function in Normal Pregnancy. Hypertension.

[25] Staff A., Zeisler H., Llurba E., Chantraine F., Vatishe M., Sennströmf M., Olovssong M., Brennecke S., Stepani H., Allegranza D. (2017). Angiogenic factors and prediction of adverse pregnancy outcomes in suspected preeclampsia: The PROGNOSIS study. Pregnancy Hypertens. Int. J. Women’s Cardiovasc. Health.

[26] Nicolaides K., Rizzo G., Hecher K., Ximenes R. (2002). Doppler in Obstetrics.

[27] Montano L., Maugeri A., Volpe M.G., Micali S., Mirone V., Mantovani A., Navarra M., Piscopo M. (2022). Mediterranean Diet as a Shield against Male Infertility and Cancer Risk Induced by Environmental Pollutants: A Focus on Flavonoids. Int. J. Mol. Sci..

[28] Lettieri G., D’Agostino G., Mele E., Cardito C., Esposito R., Cimmino A., Giarra A., Trifuoggi M., Raimondo S., Notari T. (2020). Discovery of the Involvement in DNA Oxidative Damage of Human Sperm Nuclear Basic Proteins of Healthy Young Men Living in Polluted Areas. Int. J. Mol. Sci..

[29] Lettieri G., Mollo V., Ambrosino A., Caccavale F., Troisi J., Febbraio F., Piscopo M. (2019). Molecular effects of copper on the reproductive system of mytilus galloprovincialis. Mol. Reprod. Dev..

[30] Lettieri G., Marra F., Moriello C., Prisco M., Notari T., Trifuoggi M., Giarra A., Bosco L., Montano L., Piscopo M. (2020). Molecular Alterations in Spermatozoa of a Family Case Living in the Land of Fires—A First Look at Possible Transgenerational Effects of Pollutants. Int. J. Mol. Sci..

[31] American College of Obstetricians and Gynecologists (2020). Practice Bulletin. Gestational Hypertension and Preeclampsia. Obstet. Gynecol..

[32] Sibai B., Dekker G., Kupferminc M. (2005). Pre-eclampsia. Lancet.

[33] Redman C. (2014). The six stages of pre-eclampsia. Pregnancy Hypertens. Int. J. Women’s Cardiovasc. Health.

[34] Peral J.H., Rodríguez S.M., Ayala A.U., González-Mesa E., Garcia E.S. (2013). Manejo perinatal en el límite de la viabilidad. Propuestas de abordaje en un hospital terciario. Prog. Obstet. Ginecol..

[35] Hutcheon J.A., Lisonkova S., Joseph K.S. (2011). Epidemiology of pre-eclampsia and the other hypertensive disorders of pregnancy. Best Pract. Res. Clin. Obstet. Gynaecol..

[36] Perry H., Gutierrez J., Binder J., Thilaganathan B., Khalil A. (2020). Maternal arterial stiffness in hypertensive pregnancies with and without small-for-gestational-age neonate. Ultrasound Obstet. Gynecol..

[37] Cooper L.L., Mitchell G.F. (2019). Incorporation of Novel Vascular Measures into Clinical Management: Recent Insights from the Framingham Heart Study. Curr. Hypertens. Rep..

[38] Tenório M.B., Ferreira R.C., Moura F.A., Bueno N.B., Goulart M.O.F., Oliveira A.C.M. (2018). Oral antioxidant therapy for prevention and treatment of preeclampsia: Meta-analysis of randomized controlled trials. Nutr. Metab. Cardiovasc. Dis..

[39] Kharb S. (2000). Low whole blood glutathione levels in pregnancies complicated by preeclampsia and diabetes. Clin. Chim. Acta.

[40] Nuttall S.L., Khan J.N., Thorpe G.H., Langford N., Kendall M.J. (2003). The impact of therapeutic doses of paracetamol on serum total antioxidant capacity. J. Clin. Pharm. Ther..

[41] Allegaert K., van den Anker J.N. (2017). Perinatal and neonatal use of paracetamol for pain relief. Semin. Fetal Neonatal Med..

[42] Zusterzeel P.L.M., Mulder T.P.J., Peters W.H.M., Wiseman S.A., Steegers E.A.P. (2000). Plasma protein carbonyls in nonpregnant, healthy pregnant and preeclamptic women. Free Radic. Res..

[43] Vanderlelie J., Venardos K., Clifton V.L., Gude N.M., Clarke F.M., Perkins A.V. (2005). Increased biological oxidation and reduced anti-oxidant enzyme activity in pre-eclamptic placentae. Placenta.

[44] Padmini E., Lavanya S., Uthra V. (2009). Preeclamptic placental stress and over expression of mitochondrial HSP70. Clin. Chem. Lab. Med..

[45] Llurba E., Gratacós E., Martín-Gallán P., Cabero L., Dominguez C. (2004). A comprehensive study of oxidative stress and antioxidant status in preeclampsia and normal pregnancy. Free Radic. Biol. Med..

[46] Rashid C.S., Bansal A., Simmons R.A. (2018). Oxidative Stress, Intrauterine Growth Restriction, and Developmental Programming of Type 2 Diabetes. Physiology.

[47] Aouache R., Biquard L., Vaiman D., Miralles F. (2018). Oxidative Stress in Preeclampsia and Placental Diseases. Int. J. Mol. Sci..

[48] Burton G.J., Yung H.-W., Cindrova-Davies T., Charnock-Jones D.S. (2009). Placental Endoplasmic Reticulum Stress and Oxidative Stress in the Pathophysiology of Unexplained Intrauterine Growth Restriction and Early Onset Preeclampsia. Placenta.

[49] Karowicz-Bilińska A., Suzin J., Sieroszewski P. (2002). Evaluation of oxidative stress indices during treatment in pregnant women with intrauterine growth retardation. Med. Sci. Monit..

[50] Cuffe J.S., Xu Z.C., Perkins A.V. (2017). Biomarkers of oxidative stress in pregnancy complications. Biomark. Med..

[51] Al-Kuraishy H.M., Al-Gareeb A.I., Al-Maiahy T.J. (2018). Concept and connotation of oxidative stress in preeclampsia. J. Lab. Physicians.

[52] Biri A., Bozkurt N., Turp A., Kavutcu M., Himmetoglu Ö., Durak İ. (2007). Role of Oxidative Stress in Intrauterine Growth Restriction. Gynecol. Obstet. Investig..

[53] Tamás G., Kerényi Z. (2001). Gestational diabetes: Current aspects on pathogenesis and treatment. Exp. Clin. Endocrinol. Diabetes.

[54] Zhu Y., Zhang C. (2016). Prevalence of Gestational Diabetes and Risk of Progression to Type 2 Diabetes: A Global Perspective. Curr. Diabetes Rep..

[55] National Institute for Health and Care Excellence (NICE) (2020). Diabetes in Pregnancy: Management from Preconception to the Postnatal Period.

[56] Hebert J.F., Myatt L. (2021). Placental mitochondrial dysfunction with metabolic diseases: Therapeutic approaches. Biochim. Biophys. Acta (BBA)—Mol. Basis Dis..

[57] Sisay M., Edessa D., Ali T., Mekuria A.N., Gebrie A. (2020). The relationship between advanced glycation end products and gestational diabetes: A systematic review and meta-analysis. PLoS ONE.

[58] Chen W., Zhang Y., Yue C., Ye Y., Chen P., Peng W., Wang Y. (2017). Accumulation of Advanced Glycation End Products Involved in Inflammation and Contributing to Severe Preeclampsia, in Maternal Blood, Umbilical Blood and Placental Tissues. Gynecol. Obstet. Investig..

[59] Xia L., Wang H., Munk S., Kwan J., Goldberg H.J., Fantus I.G., Whiteside C.I. (2008). High glucose activates PKC-ζ and NADPH oxidase through autocrine TGF-β 1 signaling in mesangial cells. Am. J. Physiol.-Ren. Physiol..

[60] Leloup C., Tourrel-Cuzin C., Magnan C., Karaca M., Castel J., Carneiro L., Colombani A.L., Ktorza A., Casteilla L., Pénicaud L. (2009). Mitochondrial Reactive Oxygen Species Are Obligatory Signals for Glucose-Induced Insulin Secretion. Diabetes.

[61] Kapustin R., Chepanov S., Kopteeva E., Arzhanova O. (2020). Maternal serum nitrotyrosine, 8-isoprostane and total antioxidant capacity levels in pre-gestational or gestational diabetes mellitus. Gynecol. Endocrinol..

[62] Walsh S.W. (2000). Placental isoprostane is significantly increased in preeclampsia. FASEB J..

[63] Chiarello D.I., Abad C., Rojas D., Toledo F., Vázquez C.M., Mate A., Sobrevia L., Marín R. (2020). Oxidative stress: Normal pregnancy versus preeclampsia. Biochim. Biophys. Acta (BBA)—Mol. Basis Dis..

[64] Higashi Y., Sukhanov S., Anwar A., Shai S.-Y., Delafontaine P. (2010). IGF-1, oxidative stress and atheroprotection. Trends Endocrinol. Metab..

[65] Galtier-Dereure F., Boegner C., Bringer J. (2000). Obesity and pregnancy: Complications and cost. Am. J. Clin. Nutr..

[66] Sen S., Herlihy M., Hacker M., Mcelrath T., Cherkerzian S., Oken E., Meydani S. (2019). BMI-based Prenatal Vitamins to Ameliorate Oxidative Stress in Obese Pregnant Women: A Randomized Controlled Trial (P11-135-19). Curr. Dev. Nutr..

[67] Fortuño A., Bidegain J., Baltanás A., Moreno M.U., Montero L., Landecho M.F., Beloqui O., Díez J., Zalba G. (2010). Is leptin involved in phagocytic NADPH oxidase overactivity in obesity? Potential clinical implications. J. Hypertens..

[68] Jääskeläinen T., Heinonen S., Hämäläinen E., Pulkki K., Romppanen J., Laivuori H. (2019). Impact of obesity on angiogenic and inflammatory markers in the Finnish Genetics of Pre-eclampsia Consortium (FINNPEC) cohort. Int. J. Obes..

[69] Hoch D., Gauster M., Mouzon S.H., Desoye G. (2019). Diabesity-associated oxidative and inflammatory stress signalling in the early human placenta. Mol. Asp. Med..

[70] (2020). Oficce for National Statistics, Birth Summary Tables. England and Wales. https://www.ons.gov.uk/peoplepopulationandcommunity/birthsdeathsandmarriages/livebirths/bulletins/birthsummarytablesenglandandwales/2020.

[71] Odame Anto E., Owiredu W.K.B.A., Sakyi S.A., Turpin C.A., Ephraim R.K.D., Fondjo L.A., Obirikorang C., Adua E., Acheampong E. (2018). Adverse pregnancy outcomes and imbalance in angiogenic growth mediators and oxidative stress biomarkers is associated with advanced maternal age births: A prospective cohort study in Ghana. PLoS ONE.

[72] Pandey D., Yevale A., Naha R., Kuthethur R., Chakrabarty S., Satyamoorthy K. (2021). Mitochondrial DNA copy number variation—A potential biomarker for early onset preeclampsia. Pregnancy Hypertens..

[73] Williamson R., McCarthy C., McCarthy F., Khashan A., Kenny L. (2017). OP 15 Investigating the role of mitochondrial dysfunction as a biomarker of pre-eclampsia. Pregnancy Hypertens. Int. J. Women’s Cardiovasc. Health.

[74] Duhig K.E., Myers J., Seed P.T., Sparkes J., Lowe J., Hunter R.M., Shennan A.H., Chappell L.C., on behalf of the PARROT Trial Group (2019). Placental growth factor testing to assess women with suspected pre-eclampsia: A multicentre, pragmatic, stepped-wedge cluster-randomised controlled trial. Lancet.

[75] Wikström A.-K., Nash P., Eriksson U.J., Olovsson M.H. (2009). Evidence of increased oxidative stress and a change in the plasminogen activator inhibitor (PAI)-1 to PAI-2 ratio in early-onset but not late-onset preeclampsia. Am. J. Obstet. Gynecol..

[76] Poon L.C., Nicolaides K.H. (2014). First-trimester maternal factors and biomarker screening for preeclampsia. Prenat. Diagn..

[77] Asiltas B., Surmen-Gur E., Uncu G. (2018). Prediction of first-trimester preeclampsia: Relevance of the oxidative stress marker MDA in a combination model with PP-13, PAPP-A and beta-HCG. Pathophysiology.

[78] Khalil A., Sodre D., Syngelaki A., Akolekar R., Nicolaides K.H. (2012). Maternal Hemodynamics at 11–13 Weeks of Gestation in Pregnancies Delivering Small for Gestational Age Neonates. Fetal Diagn. Ther..

[79] (2017). Zion Market Research. Global Dietary Supplements Market Will Reach USD 220 Billion in 2022. https://www.globenewswire.com/news-release/2017/01/11/905073/0/en/Global-Dietary-Supplements-Market-will-reach-USD-220-3-Billion-in-2022-Zion-Market-Research.html.

[80] Chappell L.C., Seed P.T., Briley A.L., Kelly F.J., Lee R., Hunt B.J., Parmar K., Bewley S.J., Shennan A.H., Steer P.J. (1999). Effect of antioxidants on the occurrence of pre-eclampsia in women at increased risk: A randomised trial. Lancet.

[81] Chappell L.C., Seed P.T., Kelly F.J., Briley A., Hunt B.J., Charnock-Jones D.S., Mallet A., Poston L. (2002). Vitamin C and E supplementation in women at risk of preeclampsia is associated with changes in indices of oxidative stress and placental function. Am. J. Obstet. Gynecol..

[82] González C., Claudio Vera P.G., Carvajal J. (2012). Estudio clínico randomizado del efecto de la suplementación alimenticia durante el embarazo con L-arginina y vitaminas antioxidantes en pre-eclampsia en población de alto riesgo. Rev. Chil. Obstet. Ginecol..

[83] Wang Z., Wang C., Qiu J., Ni Y., Chai S., Zhou L., Li J., Yan B., Yang J., Liu Q. (2018). The Association between Dietary Vitamin C/E and Gestational Hypertensive Disorder: A Case-Control Study. J. Nutr. Sci. Vitaminol..

[84] Piscopo M., Trifuoggi M., Scarano C., Gori C., Giarra A., Febbraio F. (2018). Relevance of arginine residues in Cu(II)-induced DNA breakage and Proteinase K resistance of H1 histones. Sci. Rep..

[85] Lorzadeh N., Kazemirad Y., Kazemirad N. (2020). Investigating the preventive effect of vitamins C and E on preeclampsia in nulliparous pregnant women. J. Perinat. Med..

[86] Korenc M., Osredkar J., Gersak K., Kumer K., Fabjan T., Sterpin S., Lucovnik M. (2020). Effect of High-Dose Intravenous Vitamin C on Postpartum Oxidative Stress in Severe Preeclampsia. Reprod. Med..

[87] Conde-Agudelo A., Romero R., Kusanovic J.P., Hassan S.S. (2011). Supplementation with vitamins C and E during pregnancy for the prevention of preeclampsia and other adverse maternal and perinatal outcomes: A systematic review and metaanalysis. Am. J. Obstet. Gynecol..

[88] Salles A.M.R., Galvao T.F., Silva M.T., Motta L.C.D., Pereira M.G. (2012). Antioxidants for Preventing Preeclampsia: A Systematic Review. Sci. World J..

[89] Rumbold A., Ota E., Hori H., Miyazaki C., Crowther C.A. (2015). Vitamin E supplementation in pregnancy. Cochrane Database Syst. Rev..

[90] Rumbold A., Ota E., Nagata C., Shahrook S., Crowther C.A. (2015). Vitamin C supplementation in pregnancy. Cochrane Database Syst. Rev..

[91] Rumbold A.R., Crowther C.A., Haslam R.R., Dekker G.A., Robinson J.S. (2006). Vitamins C and E and the Risks of Preeclampsia and Perinatal Complications. N. Engl. J. Med..

[92] Spinnato J.A., Freire S., Pinto ESilva J.L., Cunha Rudge M.V., Martins-Costa S., Koch M.A., Goco N., Santos Cde B., Cecatti J.G., Costa R. (2007). Antioxidant Therapy to Prevent Preeclampsia. Obstet. Gynecol..

[93] Villar J., Purwar M., Merialdi M., Zavaleta N., Thi Nhu Ngoc N., Anthony J., De Greeff A., Poston L., Shennan A. (2009). World Health Organisation multicentre randomised trial of supplementation with vitamins C and E among pregnant women at high risk for pre-eclampsia in populations of low nutritional status from developing countries. BJOG Int. J. Obstet. Gynaecol..

[94] Xu H., Perez-Cuevas R., Xiong X., Reyes H., Roy C., Julien P., Smith G., von Dadelszen P., Leduc L., Audibert F. (2010). An international trial of antioxidants in the prevention of preeclampsia (INTAPP). Am. J. Obstet. Gynecol..

[95] Roberts J.M., Myatt L., Spong C.Y., Thom E.A., Hauth J.C., Leveno K.J., Pearson G.D., Wapner R.J., Varner M.W., Thorp J.M. (2010). Vitamins C and E to Prevent Complications of Pregnancy-Associated Hypertension. N. Engl. J. Med..

[96] McCance D.R., Holmes V.A., Maresh M.J., Patterson C.C., Walker J.D., Pearson D.W., Young I.S. (2010). Vitamins C and E for prevention of pre-eclampsia in women with type 1 diabetes (DAPIT): A randomised placebo-controlled trial. Lancet.

[97] Hobson S.R., Gurusinghe S., Lim R., Alers N.O., Miller S.L., Kingdom J.C., Wallace E.M. (2018). Melatonin improves endothelial function in vitro and prolongs pregnancy in women with early-onset preeclampsia. J. Pineal Res..

[98] Sharma J.B., Kumar A., Kumar A., Malhotra M., Arora R., Prasad S., Batra S. (2003). Effect of lycopene on pre-eclampsia and intra-uterine growth retardation in primigravidas. Int. J. Gynecol. Obstet..

[99] Antartani R., Ashok K. (2011). Effect of lycopene in prevention of preeclampsia in high risk pregnant women. J. Turk. Ger. Gynecol. Assoc..

[100] Gao Q., Zhong C., Zhou X., Chen R., Xiong T., Hong M., Li Q., Kong M., Han W., Sun G. (2019). The association between intake of dietary lycopene and other carotenoids and gestational diabetes mellitus risk during mid-trimester: A cross-sectional study. Br. J. Nutr..

[101] Mesdaghinia E., Rahavi A., Bahmani F., Sharifi N., Asemi Z. (2017). Clinical and Metabolic Response to Selenium Supplementation in Pregnant Women at Risk for Intrauterine Growth Restriction: Randomized, Double-Blind, Placebo-Controlled Trial. Biol. Trace Elem. Res..

[102] Rayman M.P., Searle E., Kelly L., Johnsen S., Bodman-Smith K., Bath S.C., Mao J., Redman C.W. (2014). Effect of selenium on markers of risk of pre-eclampsia in UK pregnant women: A randomised, controlled pilot trial. Br. J. Nutr..

[103] Holmquist E., Brantsæter A.L., Meltzer H.M., Jacobsson B., Barman M., Sengpiel V. (2021). Maternal selenium intake and selenium status during pregnancy in relation to preeclampsia and pregnancy-induced hypertension in a large Norwegian Pregnancy Cohort Study. Sci. Total Environ..

[104] Sheikhi M., Sharifi-Zahabi E., Paknahad Z. (2017). Dietary Antioxidant Capacity and Its Association with Preeclampsia. Clin. Nutr. Res..

[105] World Health Organization (2021). World Health Organization Model List of Essential Medicines.

[106] Panagodage S., Yong H.E., Da Silva Costa F., Borg A.J., Kalionis B., Brennecke S.P., Murthi P. (2016). Low-Dose Acetylsalicylic Acid Treatment Modulates the Production of Cytokines and Improves Trophoblast Function in an in Vitro Model of Early-Onset Preeclampsia. Am. J. Pathol..

[107] Xiong W., Wang Y., Zhou X. (2021). Low-dose aspirin might alleviate the symptoms of preeclampsia by increasing the expression of antioxidative enzymes. Exp. Ther. Med..

[108] Williamson R.D., McCarthy F.P., Manna S., Groarke E., Kell D.B., Kenny L.C., McCarthy C.M. (2020). L-(+)-Ergothioneine Significantly Improves the Clinical Characteristics of Preeclampsia in the Reduced Uterine Perfusion Pressure Rat Model. Hypertension.

[109] McCarthy C., Kenny L.C. (2016). Therapeutically targeting mitochondrial redox signalling alleviates endothelial dysfunction in preeclampsia. Sci. Rep..

[110] Yang Y., Xu P., Zhu F., Liao J., Wu Y., Hu M., Fu H., Qiao J., Lin L., Huang B. (2021). The Potent Antioxidant MitoQ Protects against Preeclampsia during Late Gestation but Increases the Risk of Preeclampsia When Administered in Early Pregnancy. Antioxid. Redox Signal..

[111] Wu J., Jia J., He M.Z., Zeng Y., Zhang J.Y., Shi E.J., Lai S.Y., Zhou X., Sharifu L.M., Feng L. (2019). Placental Origins of Preeclampsia: Potential Therapeutic Targets. Curr. Med. Sci..

[112] Rumbold A., Duley L., Crowther C.A., Haslam R.R. (2008). Antioxidants for preventing pre-eclampsia. Cochrane Database Syst. Rev..

[113] Talaulikar V., Manyonda I. (2009). Vitamins C and E for the prevention of pre-eclampsia: Time to give up the ghost. BJOG Int. J. Obstet. Gynaecol..

